# IL-15 Prevents the Development of T-ALL from Aberrant Thymocytes with Impaired DNA Repair Functions and Increased NOTCH1 Activation

**DOI:** 10.3390/cancers15030671

**Published:** 2023-01-21

**Authors:** Madhuparna Nandi, Amit Ghosh, Sara Ali Akbari, Diwakar Bobbala, Marie-Josée Boucher, Alfredo Menendez, Trang Hoang, Subburaj Ilangumaran, Sheela Ramanathan

**Affiliations:** 1 Department of Immunology and Cell Biology, Faculty of Medicine and Health Sciences, Université de Sherbrooke, Sherbrooke, QC J1H 5N4, Canada; 2 Department of Medicine, Gastroenterology Unit, Faculty of Medicine and Health Sciences, Université de Sherbrooke, Sherbrooke, QC J1H 5N4, Canada; 3 Department of Microbiology and Infectious Diseases, Faculty of Medicine and Health Sciences, Université de Sherbrooke, Sherbrooke, QC J1H 5N4, Canada; 4 Institute for Research in Immunology and Cancer, University of Montreal, Montreal, QC H3C 1K3, Canada

**Keywords:** T cell acute lymphoblastic leukemia, *Scid*, IL-15, NOTCH1, MS5-DL4

## Abstract

**Simple Summary:**

Leukemia is a diverse group of hematopoietic malignancies that cause significant morbidity and mortality in children and adults. Although intensive chemotherapy can cure the majority of T-ALL cases, chemo-resistant and relapse cases have poor prognoses. Treatment of such cases requires the development of new approaches through a greater understanding of the molecular mechanisms of leukemogenesis. We have previously reported the spontaneous development of T-ALL in mice with impaired IL-15 signaling caused by IL-15 or IL-15 receptor deficiency. In this study, we examined the thymocyte developmental changes that precede leukemogenesis in these mice. Our findings reveal that IL-15 deficiency yields the expansion of aberrant TCR-negative T cells that may arise from impaired DNA repair in developing thymocytes. In addition, we show that these pre-leukemic cells display increased NOTCH1 activation and rely on survival signals provided by cytokines and growth factors, which may also be required for leukemogenesis.

**Abstract:**

We previously reported that NOD.*Scid* mice lacking interleukin-15 (IL-15), or IL-15 receptor alpha-chain, develop T-acute lymphoblastic leukemia (T-ALL). To understand the mechanisms by which IL-15 signaling controls T-ALL development, we studied the thymocyte developmental events in IL-15-deficient *Scid* mice from NOD and C57BL/6 genetic backgrounds. Both kinds of mice develop T-ALL characterized by circulating TCR-negative cells expressing CD4, CD8 or both. Analyses of thymocytes in NOD.*Scid.Il15^−/−^* mice prior to T-ALL development revealed discernible changes within the CD4^−^CD8^−^ double-negative (DN) thymocyte developmental stages and increased frequencies of CD4^+^CD8^+^ double-positive cells with a high proportion of TCR-negative CD4^+^ and CD8^+^ cells. The DN cells also showed elevated expressions of CXCR4 and CD117, molecules implicated in the expansion of DN thymocytes. T-ALL cell lines and primary leukemic cells from IL-15-deficient NOD.*Scid* and C57BL/6.*Scid* mice displayed increased NOTCH1 activation that was inhibited by NOTCH1 inhibitors and blockers of the PI3K/AKT pathway. Primary leukemic cells from NOD.*Scid.Il15^−/−^* mice survived and expanded when cultured with MS5 thymic stromal cells expressing Delta-like ligand 4 and supplemented with IL-7 and FLT3 ligand. These findings suggest that IL-15 signaling in the thymus controls T-ALL development from aberrant thymocytes with an impaired DNA repair capacity and increased NOTCH1 activation.

## 1. Introduction

T cell acute lymphoblastic leukemia (T-ALL) is an aggressive tumor that accounts for 10–15% of pediatric and 25% of adult ALL [1,2]. Although intensive chemotherapy can cure 75% of pediatric and 50% of adult T-ALL, the high proportion of chemo-resistant and relapse cases with a poor prognosis highlights the need to identify new therapeutic targets through a better understanding of the pathogenesis of T-ALL [1,2,3,4]. The activation of oncogenes and the inactivation of tumor suppressors frequently occur in human T-ALL [2,5]. While these genetic aberrations can occur as primary events, T-ALL is well recognized as having a low mutation burden in both adult and pediatric cohorts [6,7]. Emerging data indicate that the pro-neoplastic mutations of T-ALL accumulate later during disease evolution and are likely driven by other factors [4,8,9,10,11,12]. Therefore, although transgenic mice expressing oncogenes implicated in human T-ALL have immensely contributed to the understanding of the pathogenesis of this disease, there is a growing need to develop animal models to investigate how pre-leukemic cells with neoplastic potential arise and escape regulatory controls.

During intrathymic T lymphocyte development, CD4^−^CD8^−^ double-negative (DN) precursors require distinct signals for progression through the DN1 to DN4 stages and to become CD4^+^CD8^+^ double-positive (DP) cells [13,14,15]. Whereas cell proliferation and the DN1 to DN2 transition require signaling via KIT, IL-7R and NOTCH1 receptors, subsequent transition through the DN3 checkpoint selects thymocytes that express productively rearranged TCRβ, which combines with an unrearranged pre-TCRa chain to generate a signaling competent pre-TCR [16]. This ‘β-selection’ process enables pre-TCR signaling, allowing DN3 cells to traverse through the DN4 stage and become DP cells, which undergo positive and negative selection processes to generate CD4^+^ or CD8^+^ single-positive (SP) naïve T lymphocytes. T cell development is controlled by several transcription factors (TFs) that are turned on or off in a tightly regulated manner [15,17]. The aberrant activation of these TFs, such as LMO2/LYL1, HOXA, TLX1/3, NKX2 and TAL1, contributes to the pathogenesis of T-ALL [17,18,19]. Their activation mainly occurs via chromosomal translocation at the TCR locus through illegitimate recombination events that occur during the TCR-gene-rearrangement process [20,21]. Transgenic mouse models expressing these oncogenic TFs are widely used to elucidate the molecular pathogenesis of T-ALL. Other tumor-associated aberrant signaling pathways that frequently occur in human T-ALL arise from activating mutations in genes such as *NOTCH1* (>60%), *IL-7R*, *JAK/STAT*, *K–RAS* and *N–RAS,* and inactivating mutations in *PTEN* and *CDKN2A/2B* (>70%) [22,23,24].

Leukemia induction by NOTCH1 requires synergistic signaling through the pre-TCR complex, as constitutively active NOTCH1 alone does not induce leukemia in *Rag2^−/−^* or *Ptcra^−/−^* mice [25,26]. In human T-ALL, most gain-of-function mutations of *NOTCH1* occur in the homodimerization domain (HD) that results in ligand-independent activation, or in the C-terminal PEST domain that regulates ubiquitination and proteasomal degradation of the intracellular portion of NOTCH1 (ICN1, also called NICD: Notch intracellular domain), which transduces NOTCH signaling [5,23]. Up to 30% of T-ALLs carry mutations in FBXW7, which mediates the ubiquitination of ICN1 [27,28,29,30]. Activated NOTCH1 upregulates the expression of *MYC*, which promotes anabolic pathways [31,32] and cell-cycle progression [33]. Surprisingly, *NOTCH1*-activating mutations are late events in a transgenic mouse model of *TAL1*-induced T-ALL [12], whereas pre-leukemic stem cells rely on physiological NOTCH1 signaling in the thymus [34]. Indeed, a recent single-cell-analysis study suggested that chromosomal translocations and NOTCH1 activation occur later during the clonal evolution of T-ALL and that many early alterations occur in other genes that have not yet been implicated in T-ALL pathogenesis [10]. These alterations may precede many of the oncogenic genetic aberrations and contribute to their accumulation during disease evolution or relapse [4,8,9,10,11]. Together, these reports indicate that our understanding of the early events leading to T-ALL development is far from complete.

Interleukin-15 (IL-15) is a member of the IL-2 family of cytokines, which signal via the common gamma chain (γ_c_) containing receptor complexes [35]. The IL-15 receptor (IL-15R) consists of the ligand-binding IL-15Rα chain, the β chain (encoded by *Il2rb* and used by IL-2) and the common γ_c_ chain. IL-15 associates with IL-15Rα during biosynthesis and this complex is ‘*trans*-presented’ to responder cells that express the IL-15Rβγ_c_ receptor complex [36]. IL-15 is critical for the homeostasis of memory CD8^+^ T cells, natural killer (NK) cells and γδ T cells [37,38]. Loss of IL-15 signaling in thymic epithelial cells impairs the development of NKT and γδ T cells in the thymus [39]. We showed that in the *Scid* background, IL-15 deficiency promotes the development of T-ALL [40], revealing an unexpected tumor suppressor function for IL-15 in preventing the expansion of thymocytes that are otherwise destined to die in the thymus. The *Scid* mutation of the *Pkrdc* gene, which codes for the DNA-dependent protein kinase catalytic subunit required for double-strand DNA break repair by the non-homologous end joining (NHEJ) pathway, facilitates the generation of leukemic precursors due to the leakiness of the DN3 checkpoint during T cell development [41,42,43]. Although *Scid* mutation promotes T-ALL, it is not absolutely required for leukemogenesis as *Il15^−/−^* TCR transgenic (Tg) mice from a non-*Scid* background also develop T-ALL [40,44]. In the current study, we characterized the leukemic cells developing in IL-15-deficient *Scid* mice from NOD and C57BL/6 genetic backgrounds.

## 2. Materials and Methods

### 2.1. Mice

Non-obese diabetes (NOD) mice (NOD/ShiLtJ) were purchased from the Jackson Laboratory (Bar Harbor, ME, USA). NOD.*Il15^+/−^* progeny from the 11th backcross onwards were intercrossed to generate NOD.*Il15^−/−^* mice and NOD.*Il15*^+/+^ littermates [45]. IL-15 deficiency was introduced in the NOD.*Scid* mice by further backcrossing as previously described [40,45,46]. NOD.*Scid.Il15^−/−^* mice were backcrossed with NOD.*Scid* mice every two years to re-derive NOD.*Scid.Il15^−/−^* and NOD.*Scid* control lines. NOD.*Il15^−/−^* mice were crossed with NOD.*Rag1^−/−^* mice to obtain NOD.*Rag1^−/−^Il15^−/−^* and NOD.*Rag1^−/−^* control lines. C57BL/6.*Scid.Il15^−/−^* mice were generated by crossing C57BL/6.*Scid* mice (Jackson Laboratory) with C57BL/6*.Il15^−/−^* mice [46,47]. C57BL/6.*Rag1^−/−^* and C57BL/6.*Rag1^−/−^Il15^−/−^* mice were previously described [46]. All mice were maintained in sterile filter-topped cages in specific pathogen-free (SPF) facilities. The cages were changed in a laminar flow hood and were fed with an irradiated standard chow diet and water. Both male and female mice were used as the frequency and the kinetics of leukemia development were comparable in both sexes. All experiments were carried out with the approval of the Animal Care Committee of the Faculty of Medicine and Health Sciences of the Université de Sherbrooke (AEC approval number FMSS–247–2018 and 2018–2049; 2022–3594).

### 2.2. Monitoring Leukemia Development

Mice were followed for up to 8 months of age [40], and those displaying lethargy, kyphosis and piloerection were immediately sacrificed and their thymi, lymph nodes and spleens were collected. Single-cell suspensions were prepared by teasing the organs on stainless-steel meshes in phosphate-buffered saline containing 2% fetal bovine serum (PBS–2% FBS). The cells were washed with PBS–2% FBS and RBCs were lysed in ACK solution. After straining through a 70 μm nylon mesh, cells were counted and phenotyped for cell surface markers. Mice were considered leukemic when the total cellularity from the thymus and spleen exceeded 10 and 20 million, respectively, and the cells displayed the CD4^+^CD8^+^CD3*^−^*TCRαβ*^−^* phenotype [40]. Age-matched control mice were always included in the same analysis.

### 2.3. Cell Lines, Proliferation Assay and Co-Cultures

Leukemic cell lines established from the thymus, spleen or lymph node cells of leukemic NOD.*Scid.Il15^−/−^* mice (SID–T, SID–S, SID–XL, SID–XS, SID4–15L) were previously described [40]. These cells uniformly displayed a CD4^+^CD8^lo^CD3/TCR^−^ phenotype and expressed the T-ALL marker terminal deoxynucleotidyl transferase (TdT) [40]. The EL4 cell line was obtained from ATCC. Cells were plated in 96-well plates (2 × 10^4^ cells per well) in 200 μL of RPMI 1640 medium, supplemented with penicillin (10^6^ Units), streptomycin (0.1 mg/mL), 2 mM L-glutamine, 10 mM HEPES buffer, 0.1 mM non-essential amino acids, 1 μM pyruvate and 20 μM 2-mercaptoethanol and 10% heat-inactivated FCS. Cells were cultured for 72 h at 37 °C in a humidified incubator with 5% CO_2_ in the atmosphere. A total of 1 μCi of methyl-[^3^H] thymidine (NEN Life Sciences) was added per well during the last 8 h of culture. Incorporation of radioactivity was measured in a TopCount microplate scintillation counter (PerkinElmer, Woodbridge, ON, Canada). Inhibitors were added at the start of the cultures as indicated.

The thymic stromal cell line, MS5, expressing the NOTCH ligand DL4 (MS5-DL4) was previously described [44]. MS5-DL4 cells were seeded in 60 mm Petri dishes (5 × 10^4^ cells per plate) and cultured for 2 days to reach around 80% confluency (approximately 1 × 10^5^ cells per plate). These MS5-DL4 cultures were irradiated (20 Gy) before the addition of total lymph node cells (approximately 3 × 10^5^ cells per plate) from leukemic NOD.*Scid.Il15^−/−^* mice. The co-cultures were maintained in an alpha-MEM medium supplemented with 10% FBS, HEPES 10 mM, sodium pyruvate 1 mM, β-mercaptoethanol 55 µM, glutamax 2 mM and antibiotics, namely penicillin and streptomycin. In one set of cultures, FLT-3 Ligand and IL-7 (both from R&D systems; Toronto, ON, Canada at 5 ng/mL) were added. The growth medium was supplemented every 2 days.

Cell proliferation in cytokine-supplemented co-cultures was measured by 5-ethynyl-2′deoxyuridine (EdU) staining as previously described [48]. Briefly, cells were grown in coverslips and EdU (Thermo Fisher Scientific, Waltham, MA, USA; Cat # A10044) was added to the culture medium (10 μM). After 12 h, cells were washed in PBS, fixed in 4% paraformaldehyde solution, washed with 3% BSA in PBS and permeabilized using 0.3% Triton X-100 in PBS for 1 h. Incorporated EdU was revealed using the Click–iT^®^ reaction cocktail (ThermoFisher) following the manufacturer’s instructions. Briefly, 200 μL of Click-it reaction cocktail was added to each coverslip and incubated for 1 h at room temperature, followed by washing with PBS. Cells were labeled with Hoechst 33342 (ThermoFisher, 1 μg/mL) for 10 min at room temperature and coverslips were mounted on slides. The slides were examined under a confocal microscope (Olympus IX81 FV1000).

### 2.4. Flow Cytometry

Single-cell suspensions prepared from the thymus, spleen and lymph nodes were stained for flow cytometry analyses as previously described [40]. The cells were first treated with an antibody against CD16–CD32 (Fc-block; Becton Dickinson) diluted in PBS–2% FBS. After washing in PBS–2% FBS, the cells were incubated with a panel of fluorochrome-conjugated antibodies (Supplementary Table S1) diluted in the same buffer. The cells were washed, and data were acquired using the CytoFlex flow cytometer (Beckman Coulter). The data were analyzed using the FlowJo software, v 10.8 (BD Biosciences).

### 2.5. SDS-PAGE and Western Blot

Single-cell suspensions prepared from the thymi, treated or not with the NOTCH inhibitor DAPT (Sigma Aldrich, Mississauga, ON, Canada) or a PI3K inhibitor LY-294002 (Sigma Aldrich, Mississauga, ON, Canada) or CAL–101 (Selleckchem, Cederlane, Burlington, ON, Canada) as indicated, were washed with cold PBS, harvested and lysed in an SDS-PAGE sample buffer (50 mM Tris pH 6.8, 1% (*w*/*v*) SDS, 1 mM EDTA, 1 mM dithiothreitol). Equivalent amounts of proteins were separated in SDS-PAGE gels and transferred to polyvinylidene difluoride membranes. The blots were probed with primary antibodies (Supplementary Table S1), followed by incubation with the appropriate HRP-conjugated secondary antibodies and developed using the enhanced chemiluminescence reagent. Images were captured using the BioRad Geldoc system. Blots used for phosphoprotein estimation were incubated in a stripping solution (2% SDS, 62.5 mM Tris pH 6.8, 100 mM 2-mercaptoethanol) for 30 min at 55 °C, blocked and re-probed for total proteins. The intensities of the protein bands were quantified using ImageJ software v1.53t (NIH, Bethesda, MD, USA) and normalized to the corresponding values for actin as the gel-loading control for each sample.

### 2.6. RNA Extraction and RT–qPCR

Total RNA was extracted from the lymph nodes of mice using QIAzol Lysis Reagent (Qiagen, Toronto, Ontario, Canada), according to the manufacturer’s instructions. cDNA was synthetized from 1 µg of purified RNA using a QuantiTect^®^ reverse transcription kit (Qiagen, Toronto, ON, Canada). Quantitative PCR amplification reactions were carried out using the CFX Connect Real-Time PCR Detection System (Bio-Rad, Mississauga, ON, Canada) and using SYBR Green Supermix (Bio–Rad, Canada). All reactions were run in duplicate using the primers listed in Supplementary Table S2. For each sample, the raw cycle threshold (Ct) values for the genes of interest were normalized by subtracting the Ct value of the reference housekeeping gene *36B4* (ΔCt = Ct target gene − Ct Reference gene). 

### 2.7. Statistical Analyses

GraphPad Prism V9.4.1 software (San Diego, CA, USA) was used for statistical analyses and for plotting graphs.

## 3. Results

### 3.1. IL-15 Deficiency in the Scid Genetic Background Facilitates the Development of Aberrant Thymocytes Lacking the TCR 

In the spontaneous T-ALL model that we reported previously [40], loss of IL-15 or IL-15Rα in NOD mice with severe combined immunodeficiency (NOD.*Scid*) resulted in T-ALL development in all mice by 8 months of age, indicating a crucial role for IL-15 signaling in preventing leukemogenesis. As the *Scid* mutation results in impaired T cell development, we examined whether T lymphopenia caused by RAG1 deficiency can also promote spontaneous leukemia development. We also evaluated the impact of the *Scid* mutation on leukemia development in the C57BL/6 genetic background to rule out the possibility that leukemia development in IL-15-deficient *Scid* mice is dependent on NOD genetic background. Leukemia development was readily observed in all IL-15-deficient NOD.*Scid* mice and in 75% of C57BL/6.*Scid* mice, but it was not observed in IL-15-deficient NOD.*Rag1^−/−^* or C57BL/6. *Rag1^−/−^* mice (Table 1). Although both RAG1 deficiency and the *Scid* mutation of the *Pkrdc* gene impair T cell receptor (TCR)-gene-rearrangement events at the DN3 checkpoint during thymocyte development, the *Scid* mutation presents a leaky phenotype that yields the generation of leukemic precursors [41,42,43]. Hence, the frequent development of leukemia in both NOD and C57BL/6 *Scid* mice lacking IL-15 (Table 1) suggests that IL-15 controls leukemogenesis from aberrant thymocytes escaping the impaired DNA repair process.

**Figure 1:**
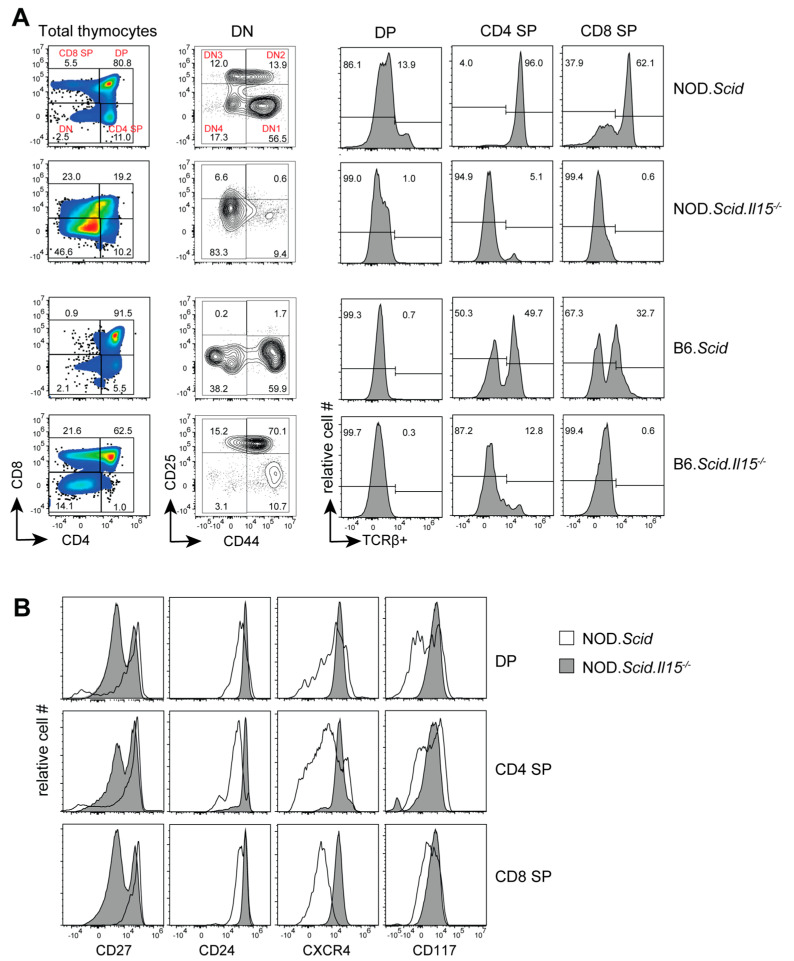
Representative flow cytometry profiles of thymocytes from leukemic NOD.*Scid.Il15^−/−^* and C57BL/6.*Scid.Il15^−/−^* and control NOD.*Scid* and C57BL/6.*Scid* mice. (**A**) Profile of thymocyte subsets based on CD4 and CD8 expression (first column) and the DN subsets based CD25 and CD44 expression (second column). Histograms (last 3 columns) show TCRβ expression in CD4 SP, CD8 SP and DP subsets. (**B**) Expression levels of CD24, CD27, CXCR4 and CD117 in CD4 SP, CD8 SP and DP subsets from NOD.*Scid* (unfilled histogram) and leukemic NOD.*Scid.Il15^−/−^* (gray histogram) mice.

To determine how IL-15 deficiency impacts T cell developmental defects caused by the *Scid* mutation, we analyzed the phenotype of thymocytes from leukemic NOD.*Scid*.*Il15^−/−^* mice and age-matched controls. NOD.*Scid*.*Il15^−/−^* mice displayed markedly different phenotypic profiles with increased frequencies of CD4^−^CD8^−^ DN thymocytes compared to NOD.*Scid* mice (Figure 1A). We observed a variable distribution of thymocyte subsets in leukemic NOD.*Scid*.*Il15^−/−^* mice (Supplementary Figure S1). Nonetheless, within the DN cell compartment, CD44^+^CD25*^−^* DN1 cells were abundant in NOD.*Scid* mice, whereas NOD.*Scid*.*Il15^−/−^* thymi displayed fewer of these cells and a higher proportion of CD44*^−^*CD25*^−^* cells. Notably, CD4^+^ SP and CD8^+^ SP cells that developed in NOD.*Scid* mice expressed the TCR whereas SP cells from leukemic NOD.*Scid*.*Il15^−/−^* thymi did not express the TCR (Figure 1A). This scenario is recapitulated in leukemic C57Bl/6.*Scid*.*Il15^−/−^* thymi (Figure 1A). Analyses of markers associated with T cell development indicated that DP, CD4^+^SP and CD8^+^SP subsets in NOD.*Scid*.*Il15^−/−^* thymi showed reduced levels of CD27 (Figure 1B), which is reported to be upregulated by pre-TCR and TCR signals [49]. On the other hand, these cells upregulated the expression of CD24, a developmental marker that is downregulated in mature SP cells (Figure 1B). These findings suggested that IL-15 deficiency yields the development of aberrant thymocytes that arise from impaired DNA repair process during TCR gene rearrangement.

**Figure 2:**
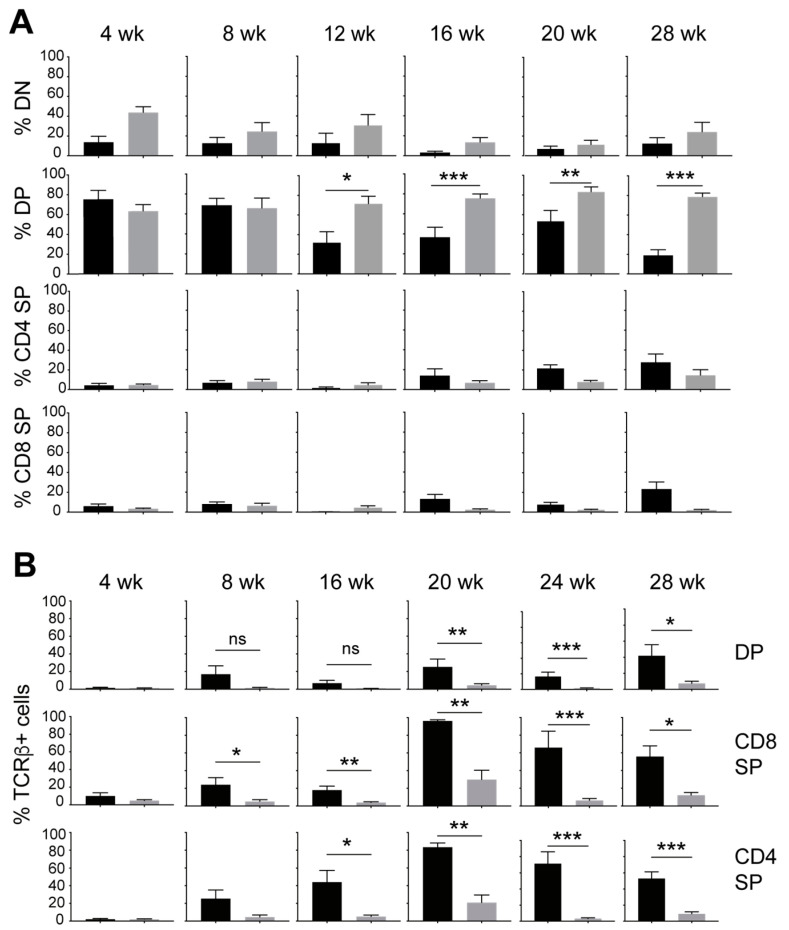
IL-15 deficiency in NOD.*Scid* mice causes an increase in the proportion of DP thymocytes while decreasing TCR-positive cells. (**A**) Frequency of DN (CD4^−^CD8^−^), DP (CD4^+^CD8^+^) and SP (CD4^+^CD8^-^, CD4^−^CD8^+^) subsets at different age groups of NOD.*Scid* (black bars) and NOD.*Scid.Il15^−/−^* (gray bars) mice. (**B**) Proportion of cells expressing TCR in DP, CD4^+^SP and CD8^+^SP subsets. *n* = 4–8 mice per group. Mean and standard deviation (SD) are shown. Statistical significance was calculated using Mann–Whitney’s test. * *p* > 0.05; ** *p* > 0.01; *** *p* > 0.001.

The development of leukemia in NOD.*Scid*.*Il15^−/−^* mice is stochastic and the incidence of T-ALL can occur from 4 weeks onwards up to 32 weeks [40]. Therefore, we characterized the aberrant thymocyte development in NOD.*Scid*.*Il15^−/−^* mice at different age groups from 4 to 28 weeks. To focus on how IL-15 deficiency deregulates T cell development prior to leukemia development, mice that had already developed leukemia were excluded. Thymocyte subset analyses based on CD4 and CD8 expression indicated that the frequency of DP thymocytes progressively increased between 12 and 28 weeks of age in NOD.*Scid.Il15^−/−^* mice when compared to the age-matched controls (Figure 2A). Next, we assessed the frequency of DP and SP thymocytes that express TCRαβ on their cell surface. At 4 weeks of age, the frequency of TCRαβ^+^ cells in DP and SP subsets were comparable between NOD.*Scid* and NOD.*Scid.Il15^−/−^* mice (Figure 2B). As the mice’s ages increase, the proportion of TCRαβ-positive cells gradually increased within CD4^+^ SP and CD8^+^ SP subsets and, to a smaller extent, in the CD4^+^CD8^+^ DP thymocytes of NOD.*Scid* mice. However, TCR expression remained low in the DP and SP thymocytes of NOD.*Scid.Il15^−/−^* mice in all age groups. These results indicate that IL-15 deficiency in the *Scid* background yields the accumulation of aberrant thymocytes that do not express TCR on the cell surface and that these abnormal thymocytes may acquire an uncontrolled growth phenotype over time to cause leukemia.

### 3.2. IL-15 Deficiency in Scid Mice Yields DN Thymocyte Progression towards the DN4 Stage with Elevated Expression of CD117 and CXCR4

**Figure 3:**
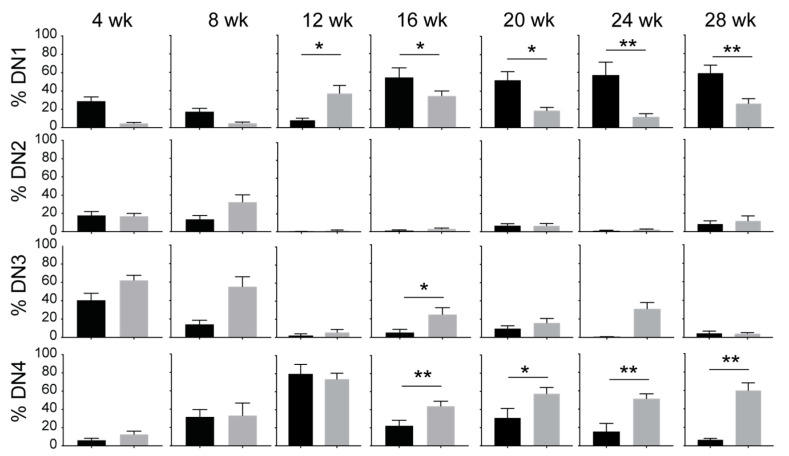
IL-15 deficiency in NOD.*Scid* mice yields the progression of DN thymocytes to the DN4 stage. Proportion of DN1 (CD44+CD25-), DN2 (CD44+CD25+), DN3 (CD44-CD25+) and DN4 (CD44-CD25-) subsets defined by CD25 and CD44 expression in NOD.*Scid* (black bars) and NOD.*Scid.Il15^−/−^* (gray bars) mice. *n* = 4–8 mice per group. Mean and standard deviation (SD) are shown. Statistical significance was calculated using Mann–Whitney’s test. * *p* > 0.05; ** *p* > 0.01.

**Figure 4:**
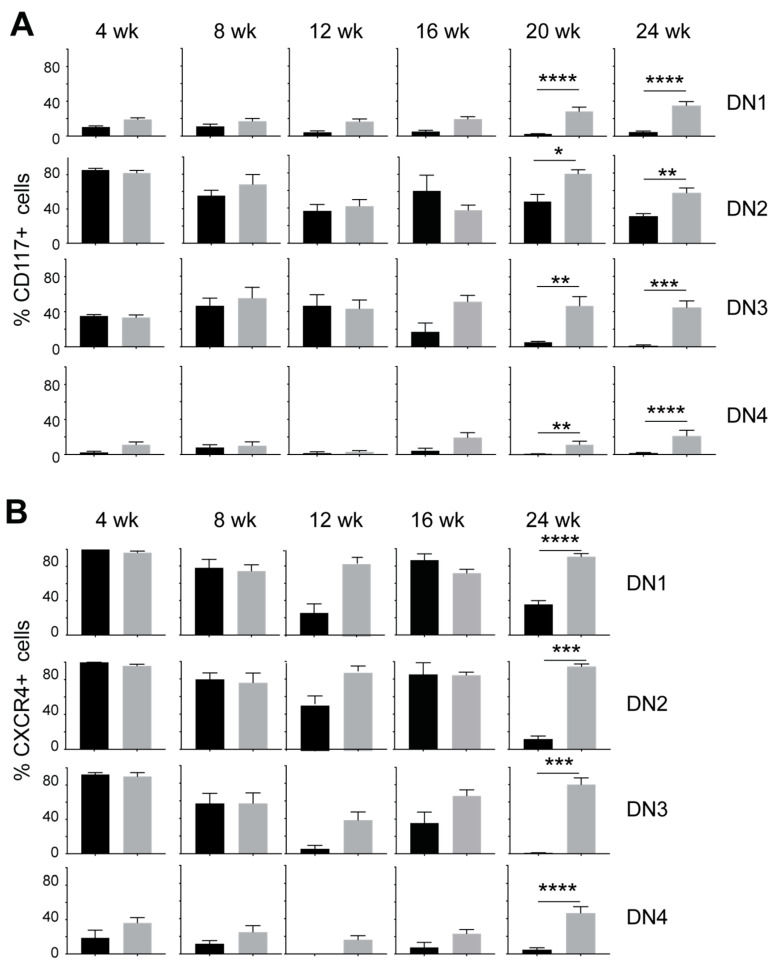
Increased expression of CD117 (cKIT) and CXCR4 in DN thymocyte subsets of IL-15-deficient NOD.*Scid* mice. Proportion of CD117^+^ (**A**) and CXCR4^+^ cells (**B**) in DN1, DN2, DN3 and DN4 subsets in NOD.*Scid* (black bars) and NOD.*Scid.Il15^−/−^* (gray bars) mice. *n* = 4–8 mice per group. Mean and standard deviation (SD) are shown. Statistical significance was calculated using Mann–Whitney’s test. * *p* > 0.05; ** *p* > 0.01; *** *p* > 0.001, **** *p* > 0.0001.

Within the DN thymocytes, the frequency of DN1 thymocytes diminished in NOD.*Scid.Il15^−/−^* mice from 16 weeks of age with a concomitant increase in DN4 thymocytes when compared to the age-matched NOD.*Scid* controls (Figure 3), suggesting that events leading to leukemogenesis likely occur within the DN developmental stage. This notion is supported by an increase in the expression of CD117 (cKIT) and CXCR4 in DP and SP cells from leukemic NOD.*Scid.Il15^−/−^* mice (Figure 1B). While CD117 promotes the stem cell factor (KIT ligand)-mediated survival and expansion of DN1 and DN2 cells but is downregulated at the DN3 stage [50], CXCR4 promotes the localization of DN3 thymocytes in the thymic cortex and contributes to their expansion [51]. An examination of the expression of CD117 and CXCR4 in DN subsets showed that both markers showed elevated expression in all DN subsets of NOD.*Scid.Il15^−/−^* mice after 16 weeks of age (Figure 4) when compared to the NOD.*Scid* controls. In contrast, the expression of CD27 was reduced in all DN subsets of NOD.*Scid.Il15^−/−^* mice, whereas the expression of CD24 was comparable in the DN subsets between the two genotypes (Supplementary Figure S2). These observations suggest that the lack of IL-15 signaling could facilitate the survival of aberrant DN cells that escaped the beta-selection process and progressed towards DP and SP cells despite lacking the TCR.

### 3.3. Leukemic Cells Originating in IL-15-Deficient Scid Mice Display Increased NOTCH1 Activation

**Figure 5:**
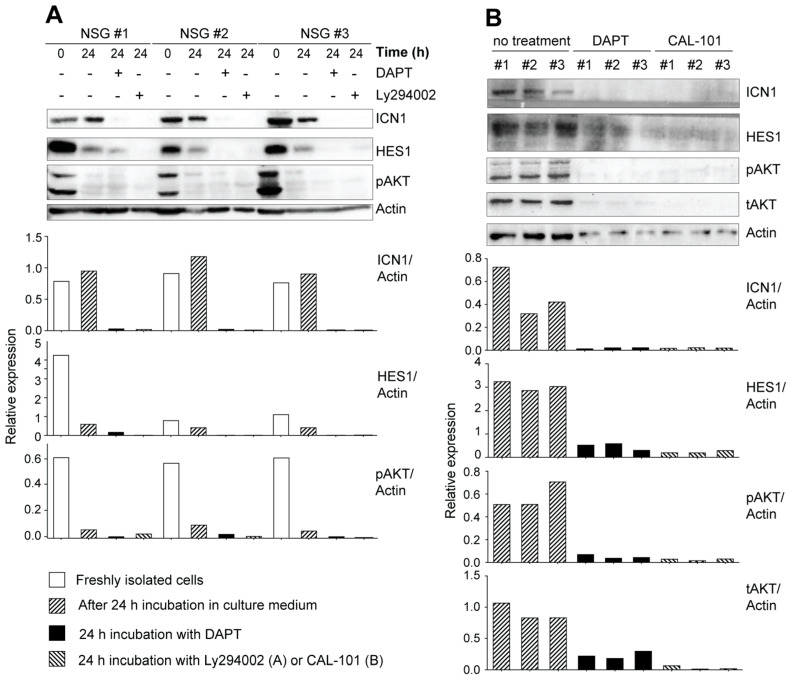
Increased NOTCH1 activation in primary leukemic cells from IL-15-deficient *Scid* mice. (**A**) Freshly isolated leukemic cells originating from a NOD.*Scid.Il15^−/−^* mouse were passaged in NSG mice to increase cell yield. Single-cell suspensions from the lymph node cells of recipient mice were lysed immediately after isolation or after 24 h incubation in the presence or absence of the gamma secretase inhibitor DAPT (10 μM) or the PI3K inhibitor LY294002 (10 μM) and analyzed for the expression of ICN1 and HES1 by Western blotting. (**B**) Leukemic cells originating from three independent C57BL/6.*Scid.Il15^−/−^* mice were incubated with DAPT or a PI3K inhibitor CAL-101 (25 μM) for 24 h and analyzed for the expression of ICN1, HES1, phospho-AKT and total-AKT. Representative data from one of the two similar experiments are shown. The densitometry quantification of ICN1, HES1, pAKT and tAKT bands normalized to the corresponding actin band for each sample is graphically represented below the blots. Original Western blots are given in Supplementary Figure S5.

Thymocyte development through the DN stages to the DP stage requires NOTCH signaling, which plays a crucial role in the development of T-ALL [52]. We observed that T-ALL cell lines established from leukemic NOD.*Scid.Il15^−/−^* or NOD.*Scid.Il15ra^−/−^* mice [40] showed constitutive expression of ICN1 and its transcriptional target HES1 (Supplementary Figure S3A). These molecules were not detectable in the EL4 thymoma cell line, which is known to express low levels of NOTCH1 [49]. In addition to DAPT, a γ-secretase inhibitor (GSI) that blocks NOTCH signaling, inhibited ICN1 generation and HES1 expression, albeit to a variable extent, in these cell lines (Supplementary Figure S3A). DAPT addition also inhibited the proliferation of these cell lines (Supplementary Figure S3B). These observations in established leukemic cell lines raise the possibility that IL-15 deficiency in NOD.*Scid* mice might yield aberrant NOTCH1 signaling in developing thymocytes, amplifying the leaky thymocyte developmental progression and leukemia development. To test this hypothesis, we first examined the expression of ICN1 and HES1 in primary leukemic cells after in vivo passage in NOD.*scid.gamma* (NSG) mice (Figure 5A). Leukemic cells originating from different NOD.*Scid.Il15^−/−^* donors expressed ICN1 and HES1 immediately after isolation that persisted for at least 24 h after in vitro culture. A similar increase in the expression of ICN1 and HES1 was observed in T-ALL cells arising from three independent IL-15-deficient C57BL/6.*Scid* mice (Figure 5B). The addition of DAPT inhibited ICN1 generation and HES1 expression in all cases. These results suggest that IL-15 deficiency promotes the emergence of T-ALL cells from aberrant thymocytes with impaired DNA repair functions and increases NOTCH1 activation.

**Figure 6:**
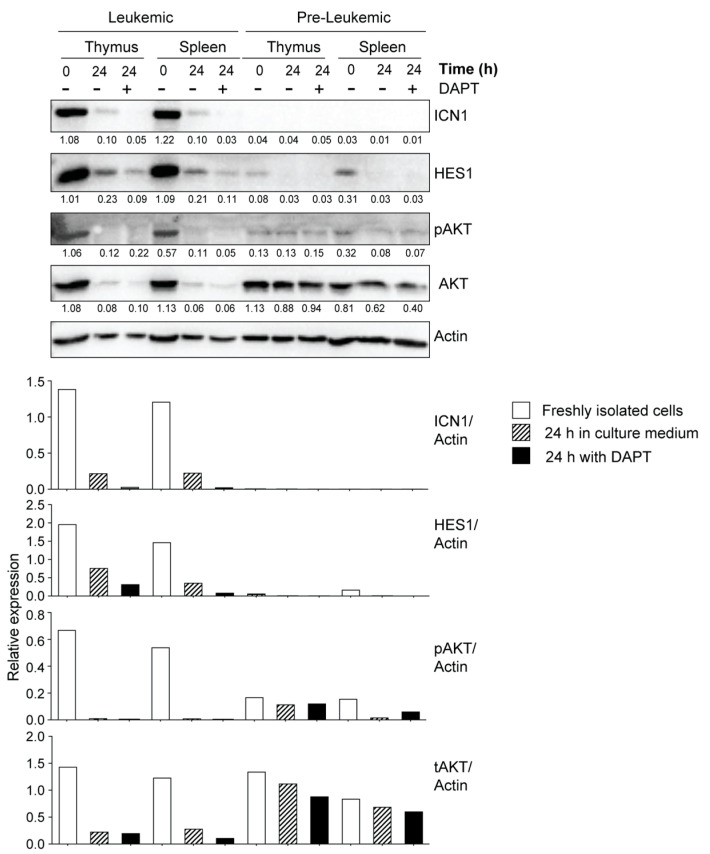
NOTCH1 expression in primary leukemic and pre-leukemic cells from IL-15-deficient *Scid* mice. Freshly isolated single-cell suspensions from thymus (pooled from at least 2–3 mice) or spleen were lysed immediately after isolation or 24 h after incubation in the presence or absence of DAPT (10 μM) and analyzed for the expression of ICN1, HES1, phospho-AKT and total-AKT. Representative data from one of the two similar experiments are shown. The densitometry quantification of ICN1, HES1, pAKT and tAKT bands normalized to the corresponding actin band for each sample is graphically represented below the blots. Original Western blots are given in Supplementary Figure S6.

Next, we assessed the expression of ICN1 and HES1 in thymocytes and splenocytes from mice that displayed increased cellularity but have not yet developed overt symptoms of leukemia, namely lethargy, kyphosis and piloerection. The yield of thymocytes from non-leukemic NOD.*Scid.Il15^−/−^* mice is generally 1–2 million cells, which is similar to cell yield from control NOD.*Scid* mice and is very limiting for protein expression analyses. As leukemia development is a stochastic event that can occur at any time point, 12–16 weeks old, asymptomatic NOD.*Scid.Il15^−/−^* mice that showed increased thymic cellularity (2–10 million) were designated ‘pre-leukemic’, and cells from these mice were used for protein expression analyses. We assessed the expression levels of ICN1 and HES1 in thymocytes and splenocytes of these ‘pre-leukemic’ mice and compared them with cells from leukemic NOD.*Scid.Il15^−/−^* mice. Although ICN1 expression was not detectable in pre-leukemic cells, low levels of HES1 could be detected in thymocytes and splenocytes ex vivo that disappeared after in vitro culture (Figure 6). These results suggest that the HES1-mediated activation of NOTCH1 target genes may occur in pre-leukemic cells.

Next, we determined whether the leukemic cells arising in NOD.*Scid.Il15^−/−^* mice displayed increased expressions of NOTCH1 target genes by real-time quantitative PCR analyses (Supplementary Figure S4). Due to the lack of a comparable cell population in the NOD.*Scid* control mice, the data are expressed as ∆Ct values with respect to the housekeeping gene *Rplp0*, which showed a Ct value of 17 that was stable in all the 12 primary leukemic samples tested. The *Cd4* gene was abundantly expressed in all samples, reflecting the DP phenotype of leukemic cells. Consistent with NOTCH1 expression and activation, *Notch1* and *cMyc* genes were abundantly expressed in all primary leukemia samples. These cells also showed abundant expression of *Tox*, which collaborates with NOTCH1 in leukemogenesis and certain members of the *Gimap* gene family that are implicated in T-ALL [53,54,55,56]. On the other hand, several other T-ALL-associated transcription factors (*Lyl1, Lmo1, Lmo2, Hoxa13, Tal1 and Tal2*) [18,21,34,57,58,59,60,61,62,63,64,65] showed variable expressions (Supplementary Figure S4). These data suggest that an increased expression of ICN1 and HES1 in the leukemic cells of NOD.*Scid.Il15^−/−^* mice is associated with the expression of NOTCH1 target genes and that their protein products likely contribute to leukemogenesis.

### 3.4. Potential Crosstalk between NOTCH and PI3K/AKT Pathways in Leukemia Development in NOD.Scid.Il15^−/−^ Mice

The PI3K/AKT signaling pathway, which synergizes with NOTCH activation to promote T cell development, can confer resistance to gamma secretase inhibitors in T-ALL [66]. Elevated basal levels of phospho-AKT in primary leukemic cells from NOD.*Scid.Il15^−/−^* and C57BL/6.*Scid.Il15^−/−^* mice (Figure 5A,B) suggested that these cells relied on both the NOTCH and PI3K/AKT pathways for leukemogenesis. The rapid reduction in pAKT levels with a discernible decrease in actin levels within 24 h of culture (Figure 5A) indicates that the primary leukemic cells require survival signals that are unavailable in the current in vitro culture conditions. At least a part of these survival signals emanates from PI3K and NOTCH1 signaling, as the inhibition of either of these pathways accelerates the reduction in actin levels (Figure 5B). We also observed that DAPT inhibited AKT activation in primary leukemic cells, but not in the pre-leukemic stage or in the established leukemic cell lines (Figure 5A,B and Figure 6, Supplementary Figure S3C). These results point towards potential crosstalk between deregulated NOTCH1 signaling and the PI3K/AKT pathway in promoting T-ALL development and a possible gain of constitutive AKT activation occurring during the establishment of cell lines in vitro.

**Figure 7:**
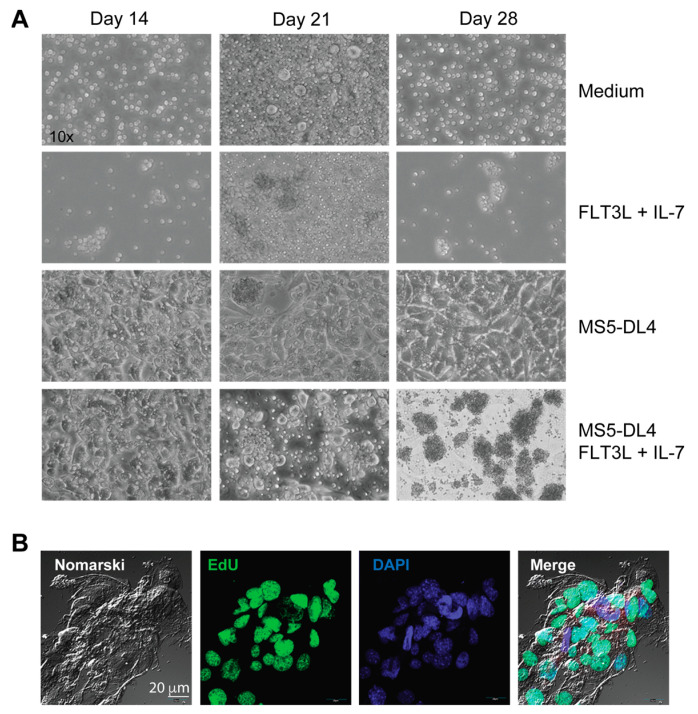
Leukemic cells maintained on MS5-DL4 cells require additional growth signals for expansion. (**A**) Phase contrast images of lymph node cells from NOD.*Scid.Il15^−/−^* mice under the indicated growth conditions. (**B**) Proliferation of leukemic cells co-cultured with irradiated MS5-DL4 cells in the presence of FLT3 ligand (FLT3L) and IL-7 was assessed by EdU staining on day 21.

In addition to deregulated NOTCH1 activation, the PI3K/AKT signaling pathway, presumably activated by cytokines and growth factors produced by thymic stromal cells in vivo, can provide survival signals to potentially leukemogenic thymocytes arising in IL-15-deficient *Scid* mice and promote the outgrowth of leukemic cells. To test whether stromal cells can sustain the growth of leukemogenic cells in vitro, we used MS5 stromal cells expressing NOTCH1 ligand Delta-like 4 (MS5-DL4), which has been shown to sustain the viability of *SCL^tg^LMO1^tg^* pre-leukemic stem cells and permit the transition of DN3 thymocytes to DP cells [67]. MS5-DL4 cultures at a confluency of 80–90% were irradiated to prevent their growth before seeding the leukemic cells isolated from NOD.*Scid.Il15^−/−^* mice. In one set of co-cultures, FLT3 ligand (FLT3L) and IL-7 were added to provide additional survival signals. Total thymocytes from NOD.*Scid* mice or non-leukemic NOD.*Scid.Il15^−/−^* mice failed to survive beyond 7 days when cultured without the MS5-DL4 feeder cells. A few leukemic cells continued to survive for up to 28 days, either alone or in the presence of IL-7 and FLT3L, although the latter cultures showed clusters of proliferating cells (Figure 7A). Co-culture with MS5-DL4 cells alone did not cause any appreciable change in cell numbers, whereas the addition of Flt3 ligand and IL–7 induced a marked proliferation of these cells as seen from marked clustering, followed by massive expansion, as observed after 28 days of culture (Figure 7A). The leukemic cells became tightly adhered to feeder cells and all cells could not be dislodged by simple pipetting, making quantitation of cell expansion difficult. However, EdU staining at day 21 after co-culture indicated that the leukemic cells were undergoing rapid proliferation (Figure 7B). These results indicate that cytokine-driven survival signals play a crucial role in expanding leukemogenic thymocytes with aberrant NOTCH1 activation that arise in IL-15-deficient *Scid* mice.

## 4. Discussion

IL-15 has been extensively studied for its indispensable roles in lymphocyte homeostasis and immune responses [68]. IL-15 improves the reconstitution of NK, NKT and CD8^+^ T cells following bone marrow transplantation in mice [69]; hence, it is considered a treatment option to boost antitumor immune responses and reduce leukemia relapse [70,71]. In contrast, IL-15 transgenic mice develop fatal NK cell leukemia, consistent with the role of IL-15 in NK cell development [72,73,74,75]. On the other hand, we reported the development of fatal T cell leukemia arising from developing thymocytes in IL-15-deficient NOD.*Scid* mice [40]. Genetic polymorphisms in IL-15 have been associated with a higher risk of T-ALL development [76,77]. However, a recent and exhaustive report on the genomic landscape of pediatric ALL that includes 2288 B-ALL and 466 T-ALL specimens revealed a total of three cases with missense mutations in *IL15RA* in B-ALL only, whereas *IL15* was the wild type [78]. Herein, we provide unambiguous genetic evidence for a functional requirement of IL-15 signaling to minimize the risk of T-ALL caused by the *Scid* mutation.

The *Scid* mutation impairs the double-stranded DNA break repair functions of PRKDC, which is required for productive TCRβ gene rearrangement, causing a developmental block at the DN3 stage [16]. However, 15–20% of the C.B-17 mice, in which the original *scid* mutation occurred, develop spontaneous thymomas (leukemia arising from pre-T cells developing in the thymus) by 60 weeks of age [79]. Transfer of the *scid* mutation to the NOD background increased thymoma incidence to 67% by 40 weeks, which has been attributed to defective thymic maturation inherent to the NOD genetic background [80,81]. While NOD.*scid* mice invariably develop thymoma [82], lymphadenopathy and splenomegaly are early features in leukemic NOD.*scid.Il15^−/−^* mice [40]. Moreover, 65% of NOD.*scid* mice develop spontaneous thymomas by 10 months of age [81], while 100% of NOD.*scid mice* lacking IL-15 or IL-15Rα develop spontaneous lymphoma/leukemia by 8 months of age [40]. Notably, the development of T-ALL in IL-15-deficient *Scid* mice is not restricted to the NOD genetic background as it also occurs in C57BL/6 mice. Although the proportion of C57BL/6.*scid.Il15^−/−^* mice developing leukemia was only 75% at 8 months of age (Table 1), our findings indicate that the loss of IL-15 signaling promotes the emergence of leukemic cells that show increased NOTCH1 activation from thymocytes harboring the *Prkcd^Scid^* mutation, irrespective of their genetic background.

Leukemic cells in NOD.*Scid.Il15^−/−^* mice express CD4 and CD8 on their cell surface, but not TCRαβ, indicating that the absence of IL-15 signaling seems to yield the survival and expansion of surface pre-TCR-negative DN3 thymocytes that proceed through the DN4 stage and become DP cells. Progression to acute leukemia is associated with an increase in DN4 and DP stage cells [12,83]. The proportion of DN1 cells, which can give rise to conventional NK cells in *Scid* mice [84,85], is decreased in pre-leukemic NOD.*Scid.Il15^−/−^*. Moreover, it is noteworthy that the transgenic expression of oncogenic TFs, such as SCL/TAL1 and TLX3, arrests T cell development at the DN3 stage when TCR genes undergo rearrangement to generate a functional receptor [12,86,87,88,89,90,91]. The progressive age-dependent accumulation of DN4 and DP thymocytes in the absence of pre-TCR/TCRb expression in NOD.*Scid.Il15^−/−^* mice is consistent with our previous work on *TAL1*-induced T-ALL, indicating that progression from a pre-leukemic DN3 stage to DP/SP8^+^ T-ALL requires the acquisition of a pre-TCR molecular signature, even in *Cd3e*-deficient mice that completely lack pre-TCR signaling [92]. Therefore, the acquisition of a post–β-selection phenotype is indicative of a molecular progression through β-selection, consistent with the importance of pre-TCR signaling for progression from a pre-leukemic stage to overt T-ALL [12,92].

The chemokine receptor CXCR4 is expressed at high levels in bone marrow precursors that seed the thymus [93]. The persistently high expression of CXCR4 observed in DN4 thymocytes from NOD.*Scid.Il15^−/−^* mice could be permissive for the leukemic cells to lodge in distal sites, such as the bone marrow, spleen and lymph nodes [40]. Mutant NOTCH1 or NOTCH3-induced T-ALLs express high levels of CXCR4 leading to their migration in response to CXCL12 [94,95]. The CXCR4/CXCL12 axis has been implicated in the infiltration of the central nervous system (CNS) and bone marrow (BM) by leukemic cells [94,96,97,98]. Vascular endothelial cells in the bone marrow environment express high levels of CXCL12 that are required for the maintenance of hematopoietic stem cells [99]. The leukemic cells may also exploit the same interaction to persist in CXCL12-rich niches [96,98]. Targeting CXCR4/CXCL12 axis in combination with JAK and BCL2 inhibitors showed diminished infiltration of the central nervous system by T-ALL in a xenograft model [100]. Whether leukemic NOD.*Scid.Il15^−/−^* mice display infiltration of the CNS and BM remains to be assessed.

T-ALL cells arising in NOD.*Scid.Il15^−/−^* mice express ICN1 and their growth is inhibited by the NOTCH1 inhibitor, DAPT. Transcripts for *c-Myc,* which is a transcriptional target of NOTCH1 signaling in leukemic cells, is detected at significant levels in primary leukemic cells from NOD.*Scid.Il15^−/−^* mice. Unlike human T-ALL cells, wherein NOTCH1 mutations occur frequently, we did not observe any mutations in the NOTCH1 heterodimerization or the PEST domains, raising the question of how NOTCH1 activation occurs in T-ALL cells from NOD.*Scid.Il15^−/−^* mice. Ligand-independent NOTCH1 activation has been reported to be caused by aberrant recombination mediated by RAG2 that generates truncated NOTCH1 [101] or the activation of a cryptic internal promoter in Ikaros-deficient cells [102], both generating a functional ICN1 that is constitutively active, leading to the development of T-ALL. Although the mechanisms underlying constitutive NOTCH1 activation in T-ALL cells of NOD.*Scid.Il15^−/−^* mice remain unclear, it appears that additional signaling pathways that activate the PI3K/AKT pathway allow for these cells to survive and expand, which would enable the acquisition of additional neoplastic characteristics leading to T-ALL. Further investigations are needed to define the IL-15-dependent regulatory processes that control the aberrant activation of the NOTCH1 and PI3K/AKT pathways that could lead to leukemogenesis of T-ALL.

In general, in vitro cultures of primary T-ALL cells is challenging; thus, they are routinely propagated in immunodeficient mice [103]. The establishment of T-ALL cell lines from primary tumors in mice also remains challenging with variable success [40,102]. Our findings indicate that the primary leukemic cells require survival signals that are unavailable in standard culture conditions (Figure 5A,B). This limitation, which constrains the study of leukemogenic signaling pathways in primary leukemic cells in vitro, could be overcome by expanding primary leukemic cells on MS5-DL4 cells in the presence of cytokines without the need for initial in vivo passaging. Future experiments using an optimized in vitro culture system are needed to assess the relative contribution of PI3K and NOTCH signaling and a potential crosstalk between these two pathways in sustaining the survival of leukemogenic cells, and how these pathways are impacted by IL-15 signaling.

## 5. Conclusions

**Figure 8:**
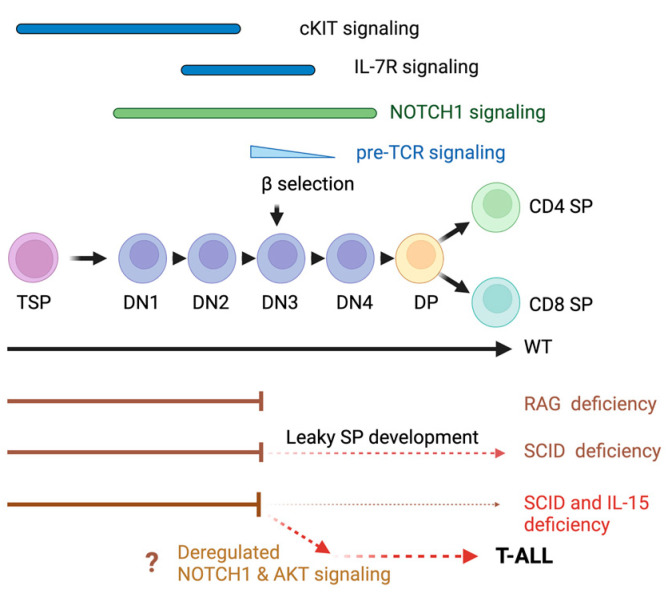
T-ALL leukemogenesis in IL-15-deficient *Scid* mice. IL-15 signaling appears to exert control over NOTCH1 and AKT signaling in the DN developmental stage to prevent the escape of potentially leukemogenic cells arising from defective DNA repair at the DN3 stage. TSP, thymic seeding progenitors. The IL-15-dependent control mechanisms remain to be elucidated.

In this study, we provide evidence for the role of IL-15 in controlling the development of T-ALL from aberrant TCR-negative thymocytes that arise from impaired DNA repair during the TCR-gene-rearrangement process (Figure 8). IL-15 deficiency appears to deregulate NOTCH1 and AKT signaling, enabling the aberrant TCR-negative thymocytes that arise in the DN3 stage to become leukemic, presumably by relieving certain IL-15-dependent control mechanisms that remain to be characterized. The ability to culture these leukemic cells on the MS5-DL4 feeder layer in the presence of cytokines will be a powerful tool to further understand how IL-15 prevents leukemogenesis.

## Figures and Tables

**Table 1:** *Scid* mutation but not RAG1 deficiency promotes leukemogenesis in the absence of IL-15.

Genotype	Leukemia Incidence ^1^	
	Males	Females
NOD.*Scid*	0/33	0/12
NOD.*Scid.Il15^−/−^*	12/12	17/17
NOD.*Rag1 ^−/−^*	0/28	0/32
NOD.*Rag1^−/−^Il15^−/−^*	0/39	0/21
C57BL/6.*Scid*	0/9	0/6
C57BL/6.*Scid.Il15^−/−^*	5/8	3/4
C57BL/6.*Rag1^−/−^*	0/29	0/46
C57BL/6.*Rag1^−/−^Il15^−/−^*	0/62	0/38

^1^ Male and female mice of the indicated genotype were followed for leukemia development for up to 8 months of age. The data were collected over a period of 4 years. Mice that displayed significant weight loss were euthanized and their thymi, spleens and lymph nodes were examined for cellularity, followed by phenotypic characterization of leukemic cells by flow cytometry. Representative flow cytometry profiles of cells from leukemic mice in NOD.*Scid* and C57BL/6.*Scid* backgrounds are shown in Figure 1. In line with the ARRIVE guidelines, the leukemic cells were used for various experiments.

## Data Availability

Not applicable.

## Supplementary Materials

The following supporting information can be downloaded at: https://www.mdpi.com/article/10.3390/cancers15030671/s1. Supplementary Table S1: Antibodies used in this study; Supplementary Table S2: RT-qPCR primers used in this study; Supplementary Figure S1: Flow cytometry profiles of thymocytes from additional leukemic NOD.*Scid.Il15^−/−^* mice; Supplementary Figure S2: Reduced expression of CD27 in mature DN thymocytes of IL-15-deficient NOD.*Scid* mice; Supplementary Figure S3: Increased NOTCH1 activation in cell lines established from NOD.*Scid.Il15^−/−^* mice; Supplementary Figure S4: Expression of leukemia-associated genes in primary leukemic cells isolated from NOD.*Scid.Il15^−/−^* mice; Supplementary Figure S5: Original Western blots for Figure 5; Supplementary Figure S6: Original Western blots for Figure 6.
