# Supplementary material for: IL-15 Prevents the Development of T-ALL from Aberrant Thymocytes with Impaired DNA Repair Functions and Increased NOTCH1 Activation
Source: Cancers (Basel). 2023 Jan 21;15(3):671. doi: 10.3390/cancers15030671 (PMC9913776; doi:10.3390/cancers15030671)
Supplement: Supplementary file 1 [file cancers-15-00671-s001.zip › cancers-2068254-supplementary.pdf]

Supplementary Table S1. Antibodies used in this study

Marker	Flurochrome	Catalog #	Source	Clone ID
CD4	APC CY5.5	MCD0419	Invitrogen	GK1.5
CD8a	eFluor450	48008182	ebiosciences	53-6.7
TCRb	PE/Dazzle594	109240	Biolegend	H57-597
CD3	PE-Cy5	15003182	eBiosciences	145-2C11
CD44	PE-Cy7	103030	Biolegend	IM7
CD25	FITC	101908	Biolegend	3C7
CD117	APC	5015056	ebiosciences	ACK2
CD27	PE	124210	Biolegend	LG.3A10
CXCR4	BV711	146517	Biolegend	L276F12
CD24	BV510	101831	Biolegend	M1/69
b-actin		CS4970	Cell Signaling	
AKT		CS9272	Cell Signaling	
p-AKT (Ser473)		CS9271	Cell Signaling	
ICN1		NEB4147	Cell Signaling	
HES1		11988S	Cell Signaling	

Supplementary Table S2. RT-qPCR primers used in this study

Gene Name	Gene ID	Sense primer	Anti-sense primer	Amplicon size (bp)
*Rplp0**	NM_007475.5	TCTGGAGGGTGTCCGCAA	CTTGACCTTTTCAGTAAGG	154
Actb	NM_007393.5	ACCTTCTACAATGAGCTGCG	CTGGATGGCTACGTACATGG	147
Myc	NM_001177353.1	GCTGTTTGAAGGCTGGATTTC	GATGAAATAGGGCTGTACGGAG	126
Cd4	NM_013488.3	GTTCGGCATGACACTCTCAG	CCTTCTCTGCCTTCCACATC	146
Tox	NM_145711	TGCTCTCCAATTCCATCTCTG	CTGTCTGATGTCTGTAGGCTG	119
Notch1	NM_145611.4	GATGAGTGTGCTCTGGGTG	TTCTGACATGGGTTGGAGATG	149
Lyl1	NM_008535	CCCTTCAGCATCTTCCCTAAC	TCACGGCTGTTGGTGAAC	119
Hoxa13	NM_008264	CCAAATGTACTGCCCCAAAG	GTTCTTTCAACTGCACCTTAGTG	149
Tal1	NM_011527	TGCCTTCCCCATGTTCAC	GCCCCATTCACATTCTGCT	150
Tal2	NM_009317	TTCACAAATACCAGGGAGCG	TGGAGGGTGAGTAGGGATAAG	87
Lmo1	NM_057173	CTCTACACCAAGGCCAACC	CCGCATCACCATCTCAAAAG	117
Lmo2	NM_008505	ACGGAAATTGTGCAGGAGAG	CGCATTTGAAACACTCCAGG	140
Gimap1	NM_008376.3	GGGACTGTCAGCAAGAAAGCTGC	CGGGGCCCTGGAGTCCTCTG	140
Gimap3	NM_031247.3	TTACAGTGTGCACCAGGAAG	CTGAATGCAACACCTCCAAC	149
Gimap4	NM_001243199.1	GGGTGAGCACCTGGGATGGGA	GCCCTGGAGAGGTCAGGGCA	126
Gimap5	NM_175035.5	AGGACATCGGAGACTGCTAC	CATGACCCCTACCCCAAAG	137

**Rplp0 (36B4)*

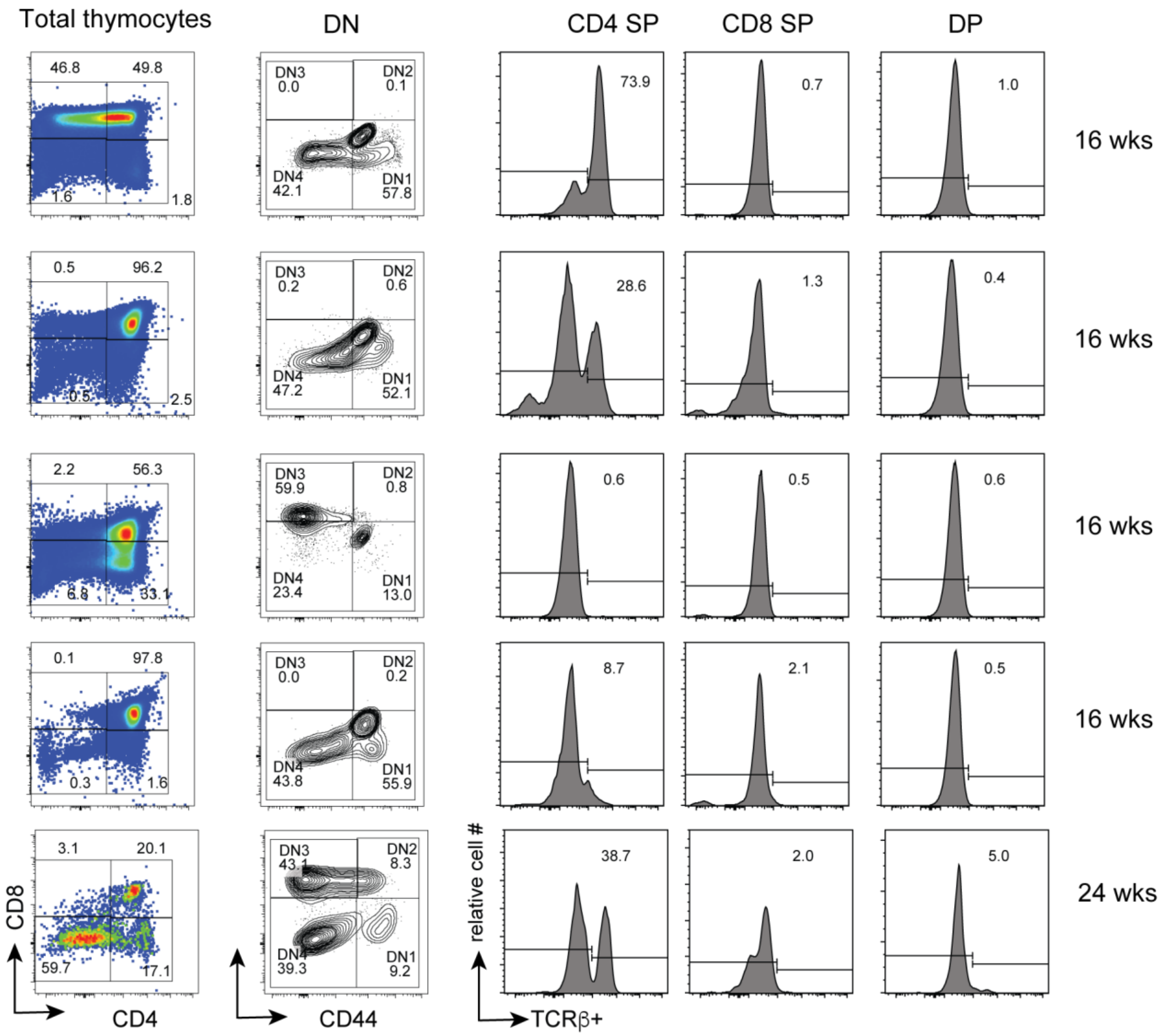

Supplementary Figure S1. Flow cytometry profiles of thymocytes from additional leukemic NOD.*Scid.Il15*$^{-/-}$ mice. Profile of thymocyte subsets based on CD4 and CD8 expression (first column) and the DN subsets based CD25 and CD44 expression (second column). Histograms (last 3 columns) show TCRβ expression in CD4 SP, CD8 SP and DP subsets.

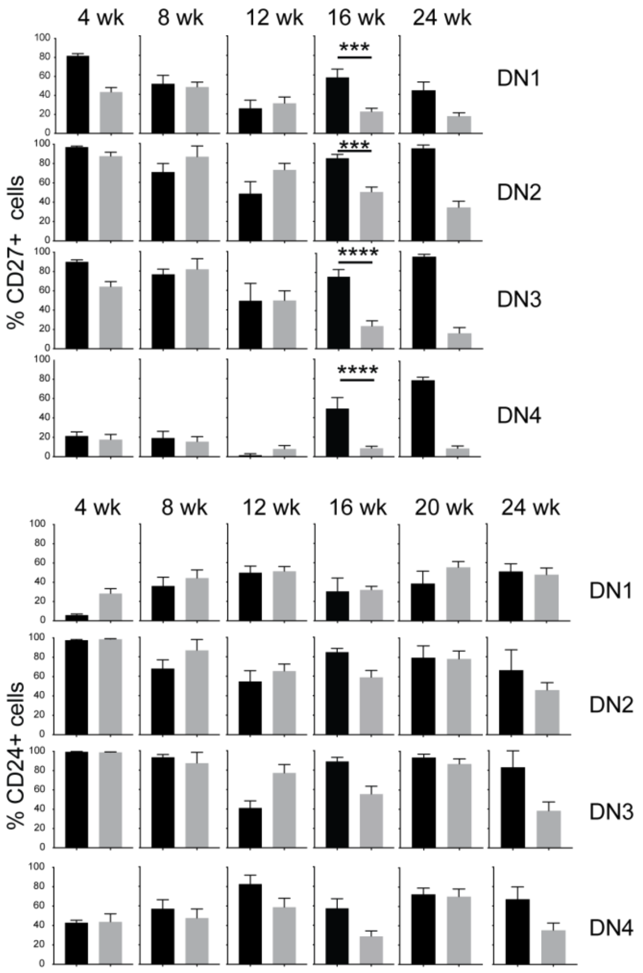

Supplementary Figure S2. Reduced expression of CD27 in mature DN thymocytes of IL-15 deficient NOD.*Scid* mice. Proportion of CD27$^+$ **(A)** and CD24$^+$ cells **(B)** in DN1, DN2, DN3 and DN4 subsets in NOD.*Scid* (Black bars) and NOD.*Scid.Il15*$^{-/-}$ (grey bars) mice. n=4-8 mice per group. Statistical significance was calculated using Mann-Whitney's test. *** p >0.001, **** p >0.0001.

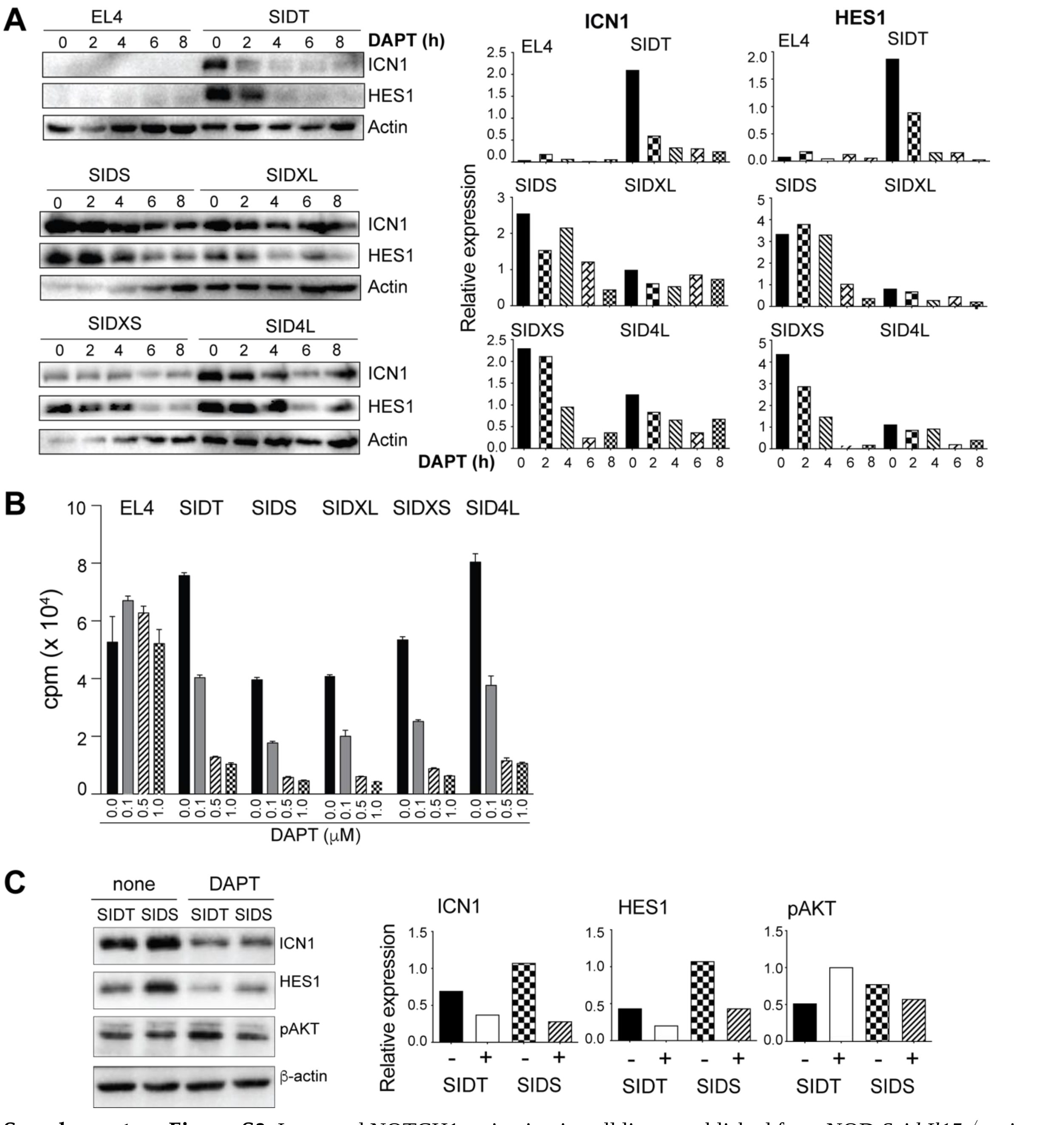

Supplementary Figure S3. Increased NOTCH1 activation in cell lines established from NOD.*Scid.Il15*$^{-/-}$ mice. (A) Indicated cell lines and EL4 controls were grown in culture in the presence of the gamma secretase inhibitor DAPT for the indicated periods of time and analyzed for the expression of ICN1 and HES1. Representative data from one of the two similar experiments are shown. Densitometry quantification of ICN1 and HES1 bands normalized to the corresponding actin band for each sample is graphically represented on the right side. (B) Inhibition of cell proliferation in T-ALL cell lines by DAPT in T-ALL cell lines. 2 x10^3 cells were seeded in 96 well plates incubated with the indicated concentration of DAPT for 32 h before adding 1 μCi of methyl–[^{3}H] thymidine for an additional 8 h. (C) NOTCH1 inhibition does not affect pAKT activation in leukemic cell lines. Indicated T-ALL cell lines were incubated with DAPT for 24 h *in vitro* and lysed to assess the expression of ICN1, HES1 and pAKT. Representative data from one of the two similar experiments are shown. Densitometry quantification of ICN1, HES1 and pAKT bands normalized to the corresponding actin band for each sample is graphically represented on the right side.

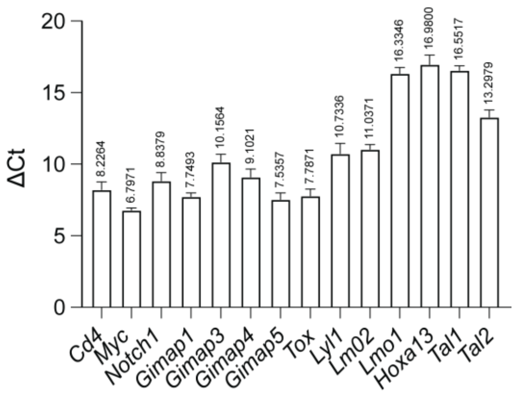

Supplementary Figure S4. Expression of leukemia-associated genes in primary leukemic cells isolated from NOD.*Scid.Il15*$^{-/-}$ mice. RNA samples extracted from total lymph node cells of 12 leukemic NOD.*Scid.Il15*$^{-/-}$ mice were evaluated individually for the expression of the indicated genes and the housekeeping gene *Rplp0* (*36b4*). The mean raw cycle threshold (Ct) values for the genes of interest were normalized by subtracting the mean Ct value of the reference gene *Rplp0* (Ct= 17.1) to obtain the mean ΔCt for each gene of interest, which is indicated above the histogram.

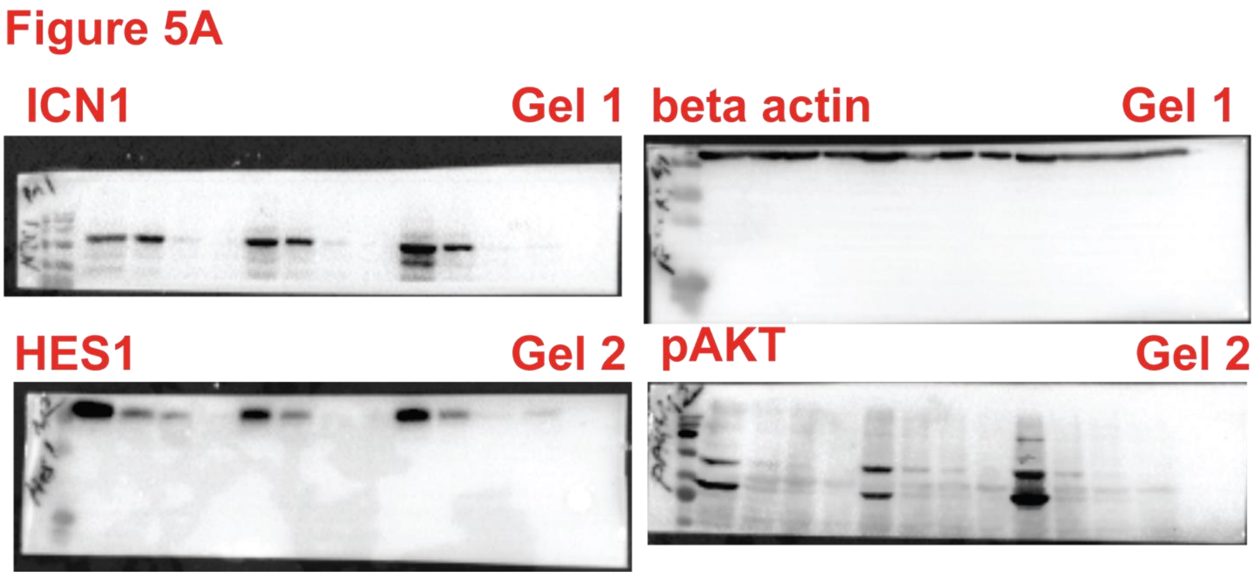

Supplementary Figure S5A. Original western blots used in Figure 5A

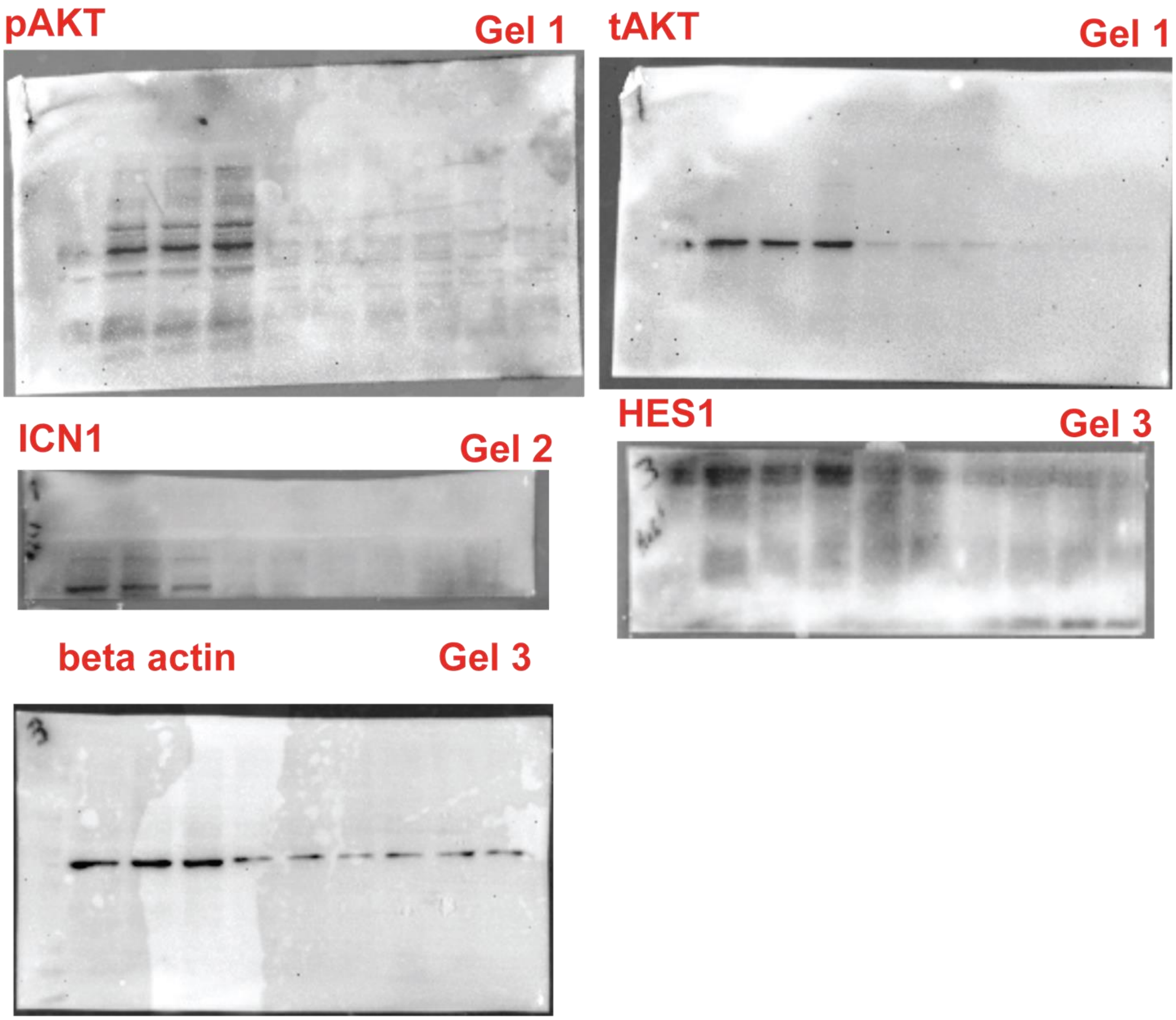

Supplementary Figure S5B. Original western blots used in Figure 5B

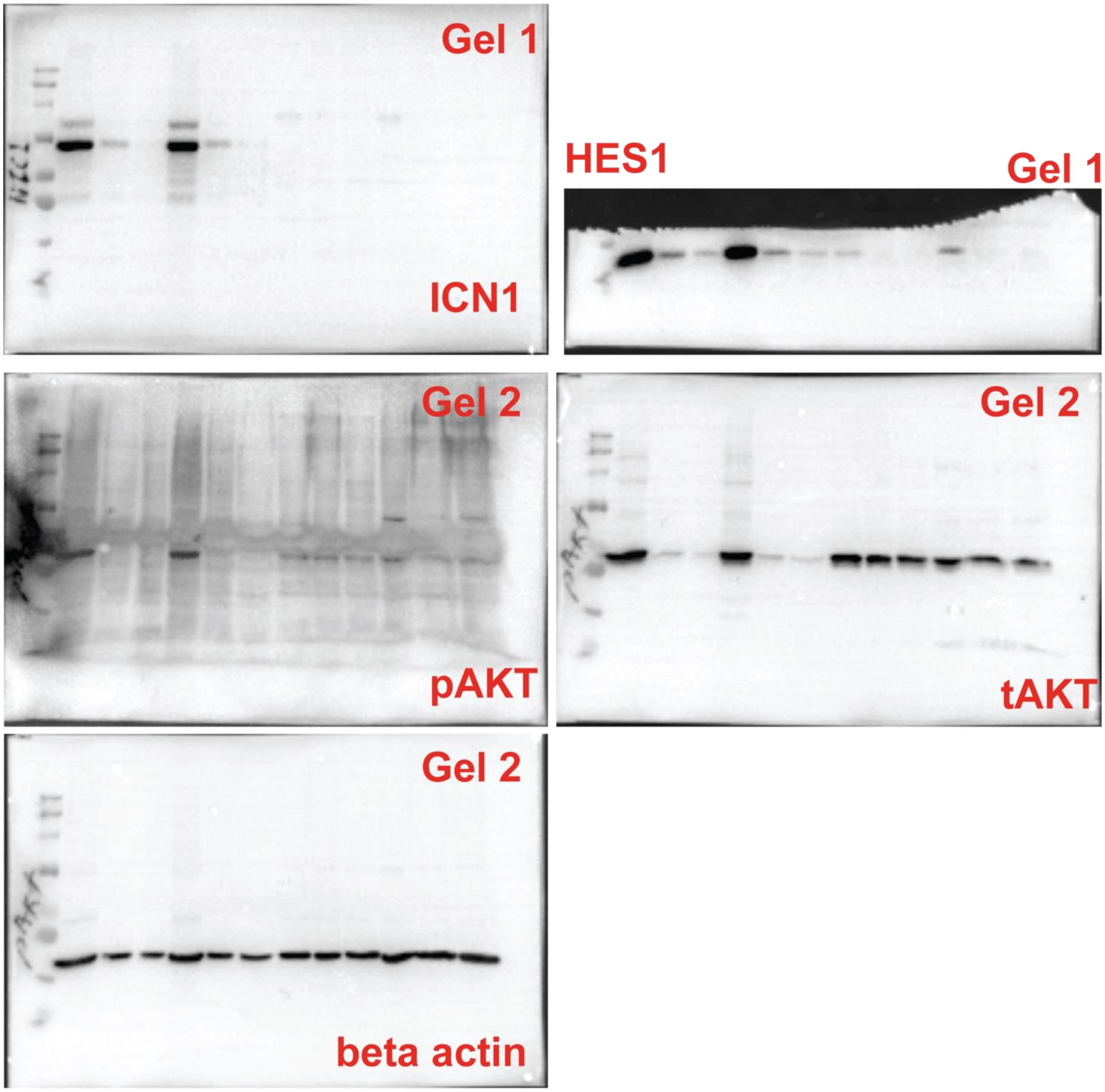

Supplementary Figure S6. Original western blots used in Figure 6.
